# Circle of Willis Variants: Fetal PCA

**DOI:** 10.1155/2013/105937

**Published:** 2013-03-21

**Authors:** Amir Shaban, Karen C. Albright, Amelia K. Boehme, Sheryl Martin-Schild

**Affiliations:** ^1^Stroke Program, Department of Neurology, Tulane University Hospital, 1440 Canal Street, TB-52, Suite 1000, New Orleans, LA 70112-2715, USA; ^2^Health Services and Outcomes Research Center for Outcome and Effectiveness Research and Education (COERE), University of Alabama at Birmingham, RWUH M226, 619 19th Street S, Birmingham, AL 35249-3280, USA; ^3^Center of Excellence in Comparative Effectiveness Research for Eliminating Disparities (CERED) Minority Health & Health Disparities Research Center (MHRC), University of Alabama at Birmingham, RWUH M226, 619 19th Street S, Birmingham, AL 35249-3280, USA; ^4^Department of Epidemiology, School of Public Health, University of Alabama at Birmingham, Birmingham, AL 35294, USA; ^5^Department of Neurology, School of Medicine, University of Alabama at Birmingham, Birmingham, AL 35294, USA

## Abstract

We sought to determine the prevalence of fetal posterior cerebral artery (fPCA) and if fPCA was associated with specific stroke etiology and vessel territory affected. This paper is a retrospective review of prospectively identified patients with acute ischemic stroke from July 2008 to December 2010. We defined complete fPCA as absence of a P1 segment linking the basilar with the PCA and partial fPCA as small segment linking the basilar with the PCA. Patients without intracranial vascular imaging were excluded. We compared patients with complete fPCA, partial fPCA, and without fPCA in terms of demographics, stroke severity, distribution, and etiology and factored in whether the stroke was ipsilateral to the fPCA. Of the 536 included patients, 9.5% (*n* = 51) had complete fPCA and 15.1% (*n* = 81) had partial fPCA. Patients with complete fPCA were older and more often female than partial fPCA and no fPCA patients, and significant variation in TOAST classification was detected across groups (*P* = 0.023). Patients with complete fPCA had less small vessel and more large vessel strokes than patients with no fPCA and partial fPCA. Fetal PCA may predispose to stroke mechanism, but is not associated with vascular distribution, stroke severity, or early outcome.

## 1. Introduction

Fetal PCA (fPCA) is a common variant of cerebral circulation. Two definitions of fPCA exist in the literature: complete fetal PCA and partial fetal PCA. Complete fetal PCA (cfPCA) is defined as posterior cerebral artery that completely originates from the internal carotid artery ICA with no connection with the basilar artery. Partial fetal PCA (pfPCA) is defined as posterior cerebral artery originating from ICA with a small, or atretic, connection with the basilar. Available studies have largely used the pfPCA definition. Variable prevalence of fPCA has been demonstrated in healthy subjects (15–32%) and those with cerebral infarction (5–36%) using autopsy or magnetic resonance angiography (MRA). Unilateral pfPCA is more frequent (11–29%) than bilateral pfPCA (1–9%). Unilateral cfPCA was detected in 4–26% of cases with only 2–4% having bilateral cfPCA. Prevalence was lower with MRA than autopsy [1]. Study population (healthy subjects versus cerebral infarction) did not influence the fPCA prevalence [1].

cfPCA can impact the anatomy of the cerebral circulation. More area is perfused by the anterior circulation as PCA is completely supplied by ICA. In addition, leptomeningeal collaterals fail to develop between the ICA and the vertebrobasilar system since both the MCA and the PCA are connected to the internal carotid system and are above the physical barrier of the tentorium while the rest of the vertebrobasilar system is below the tentorium [1, 2]. pfPCA has less impact on the vascular anatomy of the cerebral circulation: more area is perfused by the anterior circulation as PCA is mostly supplied by ICA, but the leptomeningeal collaterals may develop between anterior and posterior circulation due to the small connection that PCA has with the basilar.

Important potential consequences of this fetal variant of the circle of Willis have been demonstrated, but studies are conflicting. Increased stroke burden has been described in postmortem studies of patients with fPCA [1, 3, 4]. Another study involving living humans (*n* = 82), however, failed to demonstrate increased risk for cerebral ischemia in patients with fPCA [5]. fPCA results in left-right asymmetry on perfusion imaging which could contribute to misdiagnosis of an ischemic event, but the clinic importance of this asymmetry is yet to be determined [6]. No large study suggested that presence of fPCA may increase the likelihood of large artery stroke. However, a small exploratory study of only 18 patients with small vertebrobasilar system and fPCA described the distribution of stroke etiology with the majority of patients having large artery disease (66.7%, *n* = 12) and smaller proportions having small artery occlusions (16.7%, *n* = 3), cardioembolic (5.6%, *n* = 1), and undetermined etiology (11.1%, *n* = 2) [7]. Studies of this nature are limited and often small in sample size.

Despite fPCA being a common variant, very little is known about how this variant impacts ischemic stroke characteristics. The aim of this study was to determine if the presence of fPCA influences stroke outcome, location, severity, and etiology.

## 2. Materials and Methods

### 2.1. Patients

A retrospective analysis of prospectively identified consecutive patients who presented to our medical center with acute ischemic stroke between July 2008 and December 2010 was conducted. Patients without cerebrovascular imaging (magnetic resonance angiography (MRA) or computed tomographic angiography (CTA)) were excluded from this study. 

### 2.2. Definitions

**Figure 1:**
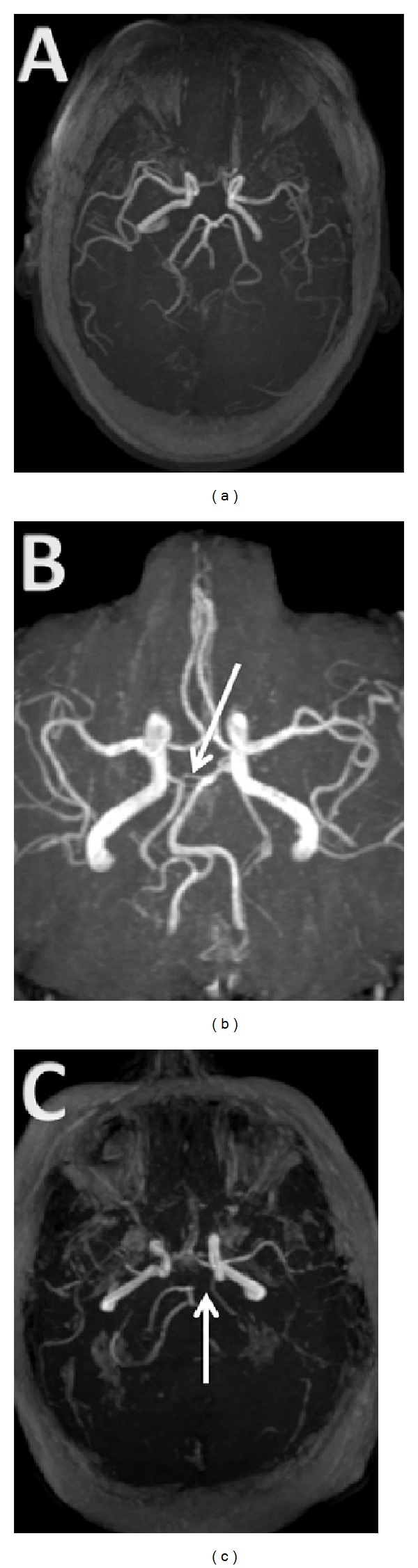
MRI TOF demonstrating a complete fetal PCA (cfPCA) and partial fetal PCA (pfPCA). (a) Typical pattern of PCAs as terminus of basilar artery. (b) Partial fPCA on the right side. The arrow shows an atretic communication between the basilar artery and the posterior communicating artery forming the PCA. (c) Complete fPCA on the left side. The arrow shows lack of communication between the basilar artery and the PCA.

We defined fPCA as cfPCA when posterior cerebral artery completely originates from the internal carotid artery ICA with no connection with the basilar artery and pfPCA when posterior cerebral artery originates from ICA with a small atretic connection with the basilar [1] (Figure 1). Patients who had cfPCA on one side and pfPCA on the other were considered as cfPCA patients. We defined another two groups of patients: patients with cfPCA on the same side of the stroke (ipsilateral cfPCA) and patients with pfPCA on the same side of the stroke (ipsilateral pfPCA). Patients with bilateral strokes were excluded from this subgroup analysis. Stroke etiology was categorized using TOAST classification [8].

### 2.3. Scans

CTA and MRA scans were interpreted by a trained stroke research fellow (AS) and reviewed for confirmation of correct assignment by a board certified vascular neurologist (SMS).

### 2.4. Statistics

Categorical data are presented as frequencies and were compared using the Pearson Chi-squared or Fisher exact test where appropriate. Continuous data are presented as medians with ranges and were compared using Wilcoxon Rank Sum test. All tests were performed at the *α* = 0.05 level and were two sided.

We compared admission demographics, initial stroke severity as measured by the baseline NIHSS, vascular distribution of infarction, and TOAST classification in patients with cfPCA, pfPCA, and no fPCA (normal cerebral circulation). Patients with cfPCA ipsilateral to their stroke were compared to patients with pfPCA ipsilateral to their stroke and to patients with fPCA without an accompanying ipsilateral stroke.

As this was an exploratory analysis, no adjustments were made for multiple comparisons [9]. The retrospective chart review was approved by the institutional review board at the Tulane University (IRB protocol number 297713-1).

## 3. Results

### 3.1. Demographics

Of the 596 patients who presented to our center, 536 patients met the inclusion criteria. The median age was 62 years old, with 41.7% (224/537) females and 65.6% (286/532) blacks. MRA was performed in 502 patients and 183 had CTA. Of the patients with complete data, 133 had both MRA and CTA performed. MRA was able to correctly identify 10 of the 11 complete fetal PCAs that were detected using CTA and correctly rule out complete fetal PCA in 121 of the 122 ruled out by CTA. Using CTA as the gold standard, the sensitivity of MRA for detecting complete fetal PCA in our sample was 90.9% (95% CI 64.4–99.2%) while specificity was 99.2% (95% CI 96.8–99.9%).

In our sample, partial fPCAs 15.1% (81/536) were more common than complete fPCAs 9.5% (51/536). Of complete fPCAs, 45.1% (23) were on the right side, 35.3% (18) were on the left side, and 19.6% (10) were bilateral. Of partial fPCAs, 43.9% (36) were on the right side, 23.3% (19) were on the left side, and 32.9% (27) were bilateral. An additional 8 patients had cfPCA on one side and pfPCA on the other. After excluding patients who experienced bilateral stroke or had evidence bilateral complete fetal PCA, we found that 38.7% (12/31) of patients with evidence of complete fetal PCA had ipsilateral stroke (not significantly different from 50.0%, *P* = 0.374). Baseline demographic information, atherosclerotic risk factors, and NIHSS at baseline were compared among patients with no fPCA, pfPCA, and cfPCA (Table 1).

Patients with complete fPCA were older (*P* = 0.025) and more commonly female (*P* = 0.013) when compared to patients with partial or no fPCA. The prevalence of vascular risk factors was similar among groups. The median NIHSS at the admission was higher for complete fPCA patients (7; range, 0–27) when compared to partial fPCA (5; range, 0–31) or no fPCA (5; range, 0–36).

### 3.2. Stroke Etiology (TOAST)

There was a significant difference among groups of the patients (*P* = 0.023). Nearly half of patients with cfPCA had large vessel etiology (40.0%, 20/50) compared to less than one-third of pfPCA patients (31.3%, 25/80), and less than one-fourth of patients with no fPCA (24%, 97/404). Patients with cfPCA had the lowest proportion of small vessel etiology (12%, 6/50) compared to pfPCA and no fPCA patients (23.8%, 19/80; 21.8%, 88/404). These results are detailed in Table 1.

After excluding patients with an acute stroke contralateral to an fPCA and those with bilateral strokes, etiology and vascular distribution of stroke were compared in patients with stroke ipsilateral to cfPCA, patients with stroke ipsilateral to pfPCA, and patients with stroke that was not ipsilateral to either cfPCA nor pfPCA (Table 2). Patients with ipsilateral cfPCA had higher frequency of large vessel disease (50%, 10/20), compared to 30.4% (14/46) of patients with ipsilateral pfPCA and 28.7% (102/356) of patients with no fPCA. There was no difference in the proportion of strokes that were ipsilateral to a cfPCA (59.0%) compared to those with stroke ipsilateral to pfPCA (66.2%). Furthermore, there was no difference in the ratio of right : left sided strokes comparing patients with cfPCA (1.3 : 1) with patients with pfPCA (1.3 : 1). Interestingly, in one of our subgroup analyses we excluded all cases of no fPCA, cases of bilateral cfPCA or pfPCA, cases of cfPCA on one side and pfPCA on the other, cases with no stroke on MRI; and cases with bilateral stroke on MRI, we end up with 70 cases, those who have cfPCA (*n* = 24) or pfPCA (*n* = 46) on a single side and detectable stroke on a single side. Strokes were ipsilateral to complete fPCA in 10/24 (41.7%) cases and ipsilateral to partial fPCA in 23/46 (50%) cases.

### 3.3. Vessel Involved

The vascular distribution of the stroke was determined in 95.7% (513/536) of patients. We found no significant difference in the involvement of MCA, PCA, or the posterior circulation comparing the no fPCA, pfPCA, and cfPCA groups of patients (Table 3).

### 3.4. Stroke Outcome

No differences were detected in any of our outcome variables including occurrence of neurological deterioration, symptomatic intracranial hemorrhage, length of stay, death, discharge modified Rankin Scale score, or discharge disposition when comparing no fPCA, pfPCA, and cfPCA, or when comparing ipsilateral cfPCA, pfPCA, and no fPCA (Table 4).

## 4. Comment

Despite fPCA being a common variant, very little is known about the prevalence and relevance of fPCA among patients with stroke. The prevalence in our population was similar to studies involving healthy subjects [1], rendering fPCA an unlikely candidate for a significant risk factor for ischemic stroke. 

We did find imbalances in patient characteristics among those with stroke and fPCA. Patients with complete fPCA were older. Since fetal PCA is a congenital variant, it could therefore be argued that a complete fPCA is protective. Similar frequencies of a prior history of stroke in patients with complete fPCA and those without fPCA argue against this. Patients with complete fPCA were more likely to be female. We found no published study providing evidence for increased frequency of complete fPCA in women in the general population. It is possible that women with complete fPCA are more susceptible to stroke, but the mechanism for such risk is unknown. We found no differences in baseline characteristics comparing patients without fPCA and those with partial fPCA.

We found a difference in the distribution of stroke etiology comparing patients with and without fPCA. Patients with cfPCA had different distribution of TOAST classification from other patients. The higher frequency of large vessel etiology and lower frequency of small vessel etiology are in keeping with the influence of lack of thalamoperforators in the absence of a P1 segment connecting the PCA with the basilar artery. We did not, however, find a higher frequency of small vessel etiology among patients with pfPCA. We expected that a small P1 segment would permit development of small thalamoperforators that would be vulnerable to atherosclerotic failure. While we found higher frequency of female patients among those with cfPCA and females may be more likely to have a small vessel disease than men [10], patients with cfPCA had lower frequency of small vessel disease.

We detected no clinical influence of the presence of fetal PCA on the outcome ischemic stroke patients. Our study expectations were based on previous literature describing the paucity of leptomeningeal collateral flow between anterior and posterior circulation, the association of the collaterals with less ischemic stroke among patients with ICA disease [11], and also the association between leptomeningeal collaterals seen on CTA and good outcome after ischemic stroke [12].

We sought to determine the frequency and implication of stroke ipsilateral to fPCA. In the presence of fPCA, we expected to find a disproportionate number of strokes ipsilateral to the fPCA. The likelihood of a stroke being ipsilateral versus contralateral to an fPCA, however, was not greater than the flip of a coin, further suggesting that the presence of fPCA does not increase the risk of ischemic stroke. Furthermore, we were unable to detect significant difference in the distribution of vascular involvement and any stroke outcome comparing patients with no fPCA, pfPCA, and cfPCA, though our sample size may have limited our power to detect such a difference.

Our results represent the largest series of patients with ischemic stroke examined for the presence and relevance of fetal PCA in patients with acute ischemic stroke. Among patients with acute ischemic stroke, the frequency of pfPCA (about 15%) and cfPCA (about 10%) was not higher than that in prior reports of persons in the general population. Similar prevalence suggests that the presence of an fPCA is not a significant risk factor for the development of ischemic stroke. Furthermore, the presence of pfPCA or cfPCA had no association with side of infarction or early clinical outcomes after infarction. We found no evidence that partial fetal PCA patients differ from patients with normal P1 segments in terms of demographics, stroke severity, etiology, and outcomes. The presence of a cfPCA, however, was associated with an increased frequency of large vessel etiology. Our study was limited by its retrospective nature and the small number of patients with stroke ipsilateral to the side of stroke to compare the distribution of vascular involvement and stroke outcomes. We acknowledge that difference on TOAST classification, age, and gender may be due to chance; a larger study needed to confirm our findings. Future studies should focus on stroke in patients with complete fetal PCA.

## Figures and Tables

**Table 1:** Demographics, vascular risk factors, and outcome in patients without fetal PCA (no fPCA), partial fetal PCA (pfPCA), and complete fetal PCA (cfPCA).

	No fPCA	pfPCA	cfPCA	*P* value
	*n* = 405	*n* = 81	*n* = 51	
Age, median years (min–max) IQR	62 (19–97) 52, 74	63 (26–93) 55, 72	74 (28–90) 57, 79	0.025
Gender, no. (female) (%)	163/405 (40.2%)	30/81 (37.0%)	31/51 (60.8%)	0.013
Race, no. (%)				0.362
White	131/403 (32.5%)	25/81 (30.9%)	8/48 (16.7%)	
Black	257/403 (63.8%)	52/81 (64.2%)	40/48 (83.3%)	
Hispanic	10/403 (2.5%)	2/81 (2.5%)	0/48 (0%)	
Other	2/403 (0.5%)	1/81 (1.2%)	0/48 (0%)	
History of prior stroke	167/402 (41.5%)	31/81 (38.3%)	22/51 (43.1%)	0.825
History of hypertension	293/399 (73.4%)	63/79 (79.7%)	39/51 (76.5%)	0.476
History of diabetes	127/400 (31.8%)	28/80 (35.0%)	13/51 (25.5%)	0.519
History of hyperlipidemia	164/398 (41.2%)	31/78 (39.7%)	25/51 (49.0%)	0.526
Active smoker	139/394 (35.3%)	30/80 (37.5%)	10/51 (19.6%)	0.066
Admission NIHSS, median (min–max) IQR	5 (0–36) 3, 12	5 (0–31) 3, 10	7 (0–27) 3, 14	0.630
Discharge NIHSS, median (min–max) IQR	2 (0–42) 1, 7	3 (0–42) 1, 9	2 (0–42) 1, 9	0.586
Discharge mRS, median (min–max) IQR	3 (0–6) 1, 4	3 (0–6) 2, 4	3 (0–6) 1.5, 4	
TOAST, no. (%)				0.023
Cardioembolic, no. (%)	97/404 (24.0%)	19/80 (23.8%)	9/50 (18.0%)	
Large vessel, no. (%)	97/404 (24.0%)	25/80 (31.3%)	20/50 (40.0%)	
Small vessel, no. (%)	88/404 (21.8%)	19/80 (23.8%)	6/50 (12.0%)	
Crypto (>1 etiology), no. (%)	8/404 (2.0%)	1/80 (1.3%)	4/50 (8.0%)	
Crypto (unknown), no. (%)	70/404 (17.3%)	9/80 (11.3)	10/50 (50.0%)	
Other, no. (%)	44/404 (10.9%)	7/80 (8.8%)	1/50 (2.0%)	

**Table 2:** TOAST classification and vessel involvement in patients without fetal PCA (no fPCA), partial fetal PCA with ipsilateral stroke (ipsilateral pfPCA), and complete fetal PCA with ipsilateral stroke (ipsilateral cfPCA).

	No fPCA	Ipsilateral pfPCA	Ipsilateral cfPCA	*P* value
	*N* = 356	*N* = 46	*N* = 20	
TOAST, no. (%)				0.621
Cardioembolic, no. (%)	73 (20.5%)	10 (21.7%)	2 (10.0%)	
Large vessel, no. (%)	102 (28.7%)	14 (30.4%)	10 (50%)	
Small vessel, no. (%)	87 (24.4%)	10 (21.7%)	3 (15.0%)	
Crypto (>1 etiology), no. (%)	7 (2.0%)	1 (2.2%)	1 (5.0%)	
Crypto (unknown), no. (%)	53 (14.9%)	6 (13.0%)	4 (20.0%)	
Other, no. (%)	34 (9.6%)	5 (10.9%)	0 (0%)	
MCA involved in the stroke	262/351 (74.6%)	38/46 (82.6%)	15/20 (75.0%)	0.497
PCA involved in the stroke	36/351 (10.3%)	3/46 (6.5%)	4/20 (20.0%)	0.253
Posterior circulation involvement	45/351 (12.8%)	7/46 (15.2%)	5/20 (25.0%)	0.289

**Table 3:** Vascular involvement of the stroke in patients without fetal PCA (no fPCA), partial fetal PCA (pfPCA), and complete fetal PCA (cfPCA).

	No fPCA	pfPCA	cfPCA	*P* value
	*N* = 387	*n* = 78	*n* = 48	
MCA involved in the stroke	269 (69.5%)	52 (66.7%)	36 (75.0%)	0.613
PCA involved in the stroke	54 (14.0%)	7 (9.0%)	8 (16.7%)	0.396
Posterior circulation involvement	68 (17.6%)	16 (20.5%)	11 (22.9%)	0.591

**Table 4:** Demographics, vascular risk factors, and outcome in patients without fetal PCA (no fPCA), ipsilateral partial fetal PCA (pfPCA), and ipsilateral complete fetal PCA (cfPCA).

	No fPCA ipsilateral to stroke	Ipsilateral pfPCA	Ipsilateral cfPCA	*P* value
	*n* = 356	*N* = 47	*N* = 20	
Age, mean (SD)	62.6 (14.7)	63.5 (13.2)	72.8 (12.5)	0.014
Female, %	38.9	44.7	55.0	0.294
Race, %				0.568
White	29.1 (103/354)	27.7 (13/47)	20 (4/20)	
Black	67.2 (238/354)	68.1 (32/47)	80 (16/20)	
Hispanic	2.5 (9/354)	0	0	
Other	0.3 (1/354)	2.1 (1/47)	0	
Admission glucose, mean (SD)	139.2 (75.6)	148.5 (98.3)	135.4 (47.6)	0.611
Baseline NIHSS, median (min–max)	5 (0–36)	7 (0–24)	5 (0–26)	0.969
Discharge NIHSS, median (min–max)	3 (0–42)	4 (0–42)	3 (0–42)	0.678
Discharge mRS, median (min–max)	3 (0–6)	3 (0–6)	3.5 (0–6)	
Discharge mRS 0–2, %	40.8	40.9	25.0	0.373
Discharge disposition				0.891
Home	165/353 (46.7%)	23/46 (50.0%)	9/20 (45.0%)	
Long term acute care	12/353 (3.4%)	3/46 (6.5%)	1/20 (5.0%)	
Inpatient rehab	119/353 (33.7%)	17/46 (37.0%)	6/20 (30.0%)	
Hospice	10/353 (2.8%)	0/46 (0%)	0/20 (0%)	
Skilled nursing	22/353 (6.2%)	2/46 (4.3%)	2/20 (10%)	
Dead	21/353 (5.9%)	1/46 (2.2%)	2/20 (10%)	
Other	4/353 (1.1%)	0/46 (0%)	0/20 (0%)	
Length of stay, median (min–max)	5.5 (1–52)	6 (1–49)	6.5 (1–44)	0.848
